# Validity of chronometric TMS for probing the time-course of word production: a modified replication

**DOI:** 10.1093/cercor/bhad081

**Published:** 2023-05-04

**Authors:** Adrian Jodzio, Vitória Piai, Lennart Verhagen, Ian Cameron, Peter Indefrey

**Affiliations:** Max Planck Institute for Psycholinguistics, Nijmegen, The Netherlands; Donders Institute for Brain, Cognition and Behaviour, Radboud University, Nijmegen, The Netherlands; Donders Institute for Brain, Cognition and Behaviour, Radboud University, Nijmegen, The Netherlands; Donders Institute for Brain, Cognition and Behaviour, Department of Medical Psychology, Radboudumc, Nijmegen, The Netherlands; Donders Institute for Brain, Cognition and Behaviour, Radboud University, Nijmegen, The Netherlands; Donders Institute for Brain, Cognition and Behaviour, Radboud University, Nijmegen, The Netherlands; Faculty of Electrical Engineering, Mathematics and Computer Science (EEMCS), University of Twente, Enschede, The Netherlands; Max Planck Institute for Psycholinguistics, Nijmegen, The Netherlands; Donders Institute for Brain, Cognition and Behaviour, Radboud University, Nijmegen, The Netherlands; Institut für Sprache und Information at Heinrich Heine University, Düsseldorf, Germany

**Keywords:** word production, response-locked analyses, discomfort ratings, peripheral effects, individual variability

## Abstract

In the present study, we used chronometric TMS to probe the time-course of 3 brain regions during a picture naming task. The left inferior frontal gyrus, left posterior middle temporal gyrus, and left posterior superior temporal gyrus were all separately stimulated in 1 of 5 time-windows (225, 300, 375, 450, and 525 ms) from picture onset. We found posterior temporal areas to be causally involved in picture naming in earlier time-windows, whereas all 3 regions appear to be involved in the later time-windows. However, chronometric TMS produces nonspecific effects that may impact behavior, and furthermore, the time-course of any given process is a product of both the involved processing stages along with individual variation in the duration of each stage. We therefore extend previous work in the field by accounting for both individual variations in naming latencies and directly testing for nonspecific effects of TMS. Our findings reveal that both factors influence behavioral outcomes at the group level, underlining the importance of accounting for individual variations in naming latencies, especially for late processing stages closer to articulation, and recognizing the presence of nonspecific effects of TMS. The paper advances key considerations and avenues for future work using chronometric TMS to study overt production.

## Introduction

Typical conversational speech unfolds at a rate of ~2 words per second. It is fast and efficient yet a highly complex and coordinated series of processes is required to produce even a single word. According to a well-supported theory (Levelt et al. 1999), the following processing stages are at the heart of word production: conceptualization, lemma selection, phonological code retrieval, syllabification, phonetic encoding, and articulation. These stages can be operationalized and studied in, for example, the context of picture naming. Here, individuals must retrieve the corresponding concept (conceptualization), select an appropriate lexical label (lemma selection), retrieve the sounds required to form this label (phonological code retrieval), put them together according to phonological rules and intonation patterns (syllabification), program an intricate series of muscles movements (phonetic encoding), and finally articulate a single word (articulation).

Building on the aforementioned theory, Indefrey and Levelt (2004) conducted a comprehensive meta-analysis, henceforth the I&L model, providing both spatial and temporal estimates for each of the proposed processing stages (Indefrey and Levelt 2004; Indefrey 2011). Although subsequent proposals for the functional neuroanatomy of language production have been put forth (Hickok 2012; Price 2012; Walker and Hickok 2016), only the I&L model provides temporal estimates for the processes underlying word production. All temporal estimates can be given relative to picture onset and to the naming latency. Assuming a naming latency of 600 ms, a common baseline naming latency for picture naming (Indefrey and Levelt 2004), they are as follows: (i) conceptualization occurring in widespread brain regions prior to 200 ms; (ii) lemma selection in the left mid portion of the middle temporal gyrus (MTG) between 200 and 275 ms; (iii) phonological code retrieval in the left posterior MTG/STG between 275 and 355 ms; (iv) syllabification in the left posterior inferior frontal gyrus (IFG, pars opercularis) between 355 and 455 ms (duration varies with number of syllables); (v) phonetic encoding in bilateral inferior motor cortices between 455 and 600 ms; and (vi) self-monitoring takes place in bilateral STG.

Although much of the literature supports the timing and brain region estimates provided by the I&L model, some contradictory findings have been reported. Strijkers and Costa (2016) have raised issues and pointed out discrepancies that need to be addressed. Among other points, they focus on the findings of a chronometric TMS study conducted by Schuhmann et al. (2012). These researchers stimulated the left mid-portion of MTG (mMTG), left posterior STG (pSTG), and left IFG in 5 time-windows postpicture onset (150, 225, 300, 400, and 500 ms). Compared with a no-stimulation condition, mMTG stimulation was found to significantly increase naming latencies when stimulated at 225–275 ms as well as at 400–450 ms, pSTG at 400–450 ms, and IFG at 300–350 ms.

Following TMS to mMTG, they observed behavioral perturbations, slowing of naming latencies, that match predictions of the I&L model. The earlier perturbation effect (225–275 ms) aligns with the lemma selection process thought to take place in mMTG, whereas the later perturbation (400–450 ms), also found after pSTG stimulation, aligns with a self-monitoring role of these regions at later stages in word production. They also observed effects of TMS interference that, on first consideration, might seem to contradict the I&L model, such as an earlier effect of IFG stimulation and no evidence of pSTG involvement (Strijkers and Costa 2016). We note, however, that alternative explanations for these apparent discrepancies are feasible. First and foremost, the earlier IFG involvement might be entirely driven by a shorter overall naming latency of the Schuhmann et al. study (460 ms) compared with the original I&L model (assuming a 600-ms latency). A concomitant reduction in processing time would explain an earlier recruitment of IFG. Furthermore, the absence of an effect for pSTG is not evidence against the I&L model. Namely, the I&L model assigns both pSTG as well as posterior middle temporal gyrus (pMTG) to phonological code retrieval. Since the pSTG stimulation site in (Schuhmann et al. 2012) was rather dorsal (Talairach: *x* = −55; *y* = −44; *z* = 18), it may be the case that TMS interfered with self-monitoring (also assigned to pSTG) but left phonological code retrieval intact.

**Fig. 1:**
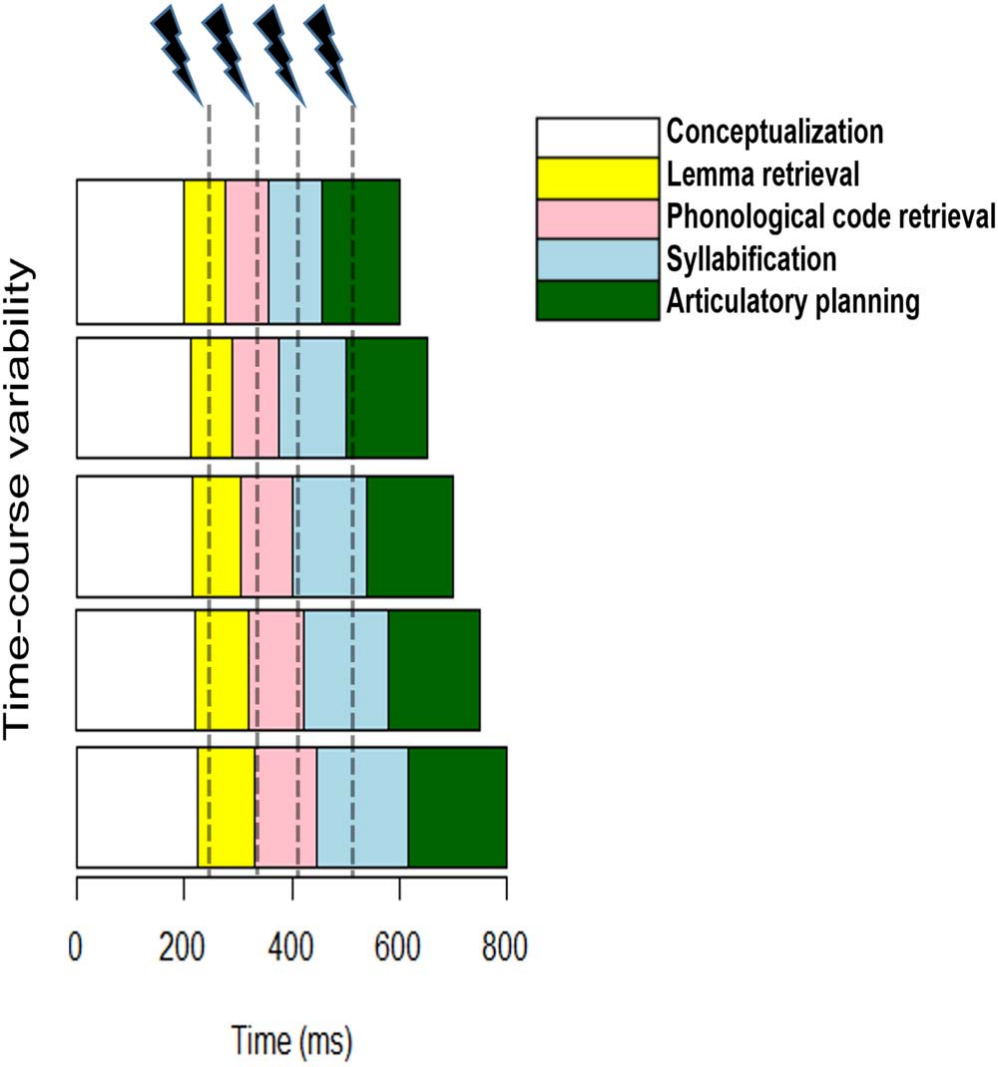
Schematic of TMS stimulation targeting varying processing stages. Each row represents a different time-course of stages dependent on naming latency. Naming latency is characterized by the end of the articulatory planning stage (green). The dashed lines indicate the timing of the TMS pulses. Each stage is illustrated by a different color as represented in the figure legend. Note that in the figure the scaling of each stage is proportionate, however this is simply to illustrate the point. It is also conceivable that longer naming latencies may reflect a longer duration in only one processing stage, in which case a different shift of processing stages might occur as a result.

In the present study, we build on and extend these findings of TMS chronometry of language production while taking the issue of naming latency variability to heart. As already mentioned for the Schuhmann et al. (2012) study, the baseline (unperturbed) naming latencies vary greatly across studies (all values are approximated): 450 ms (Schuhmann et al. 2009), 460 ms (Schuhmann et al. 2012), 525 ms (Shinshi et al. 2015), 570 ms (Zhang et al. 2018), 600 ms (Hämäläinen et al. 2018), and 620 ms (Wheat et al. 2013). If naming latencies are off by 50 ms between studies, this could mean that the targeted processing stage is also shifted in time, and thus, the same brain region being stimulated in 2 different studies may be causally contributing in 2 distinct time-windows simply as a result of differences in baseline naming latencies. Concurrently, there is also variation among participants within studies. For example, Shinshi et al. (2015) found that TMS to left IFG compared with sham stimulation significantly increased naming latencies at either 300 or 375 ms for 9 participants, at 150 ms for 1 participant, and at 225 ms for another, and no significant effects were found for a remaining participant. Again, if 1 participant names pictures 50 ms faster than another participant, TMS may perturb the target processing stage in 1 participant and leave it intact in another. This logic is illustrated in Fig. 1.

To foreshadow the methods and results, the current study also observed variability in baseline naming latencies across participants, despite preselecting stimuli to evoke naming latencies of ~600 ms. In order to address this issue, a baseline-adjusted response-locked analysis was performed. Even if there are differences in baseline naming latencies across participants, it is true that earlier processing stages may still unfold somewhat uniformly across participants. However, variation will inevitably increase as time unfurls, thus affecting later processing stages to a greater extent. By performing a baseline response-locked analysis, we may be better able to elucidate any effects arising in the later time-windows.

Lastly, TMS is also known to elicit nonspecific effects. When stimulating with TMS, anything under the TMS coil will inevitably be stimulated, this includes any muscles and nerves between the coil and the to-be stimulated cortex. TMS also elicits a “click” noise every time a pulse is triggered. Both the somatosensory and auditory by-products have been shown to evoke a-specific behavioral effects. For example, Holmes and Meteyard (2018) found that subjective discomfort ratings from different stimulation sites were able to predict reaction time differences in previously published studies. Furthermore, studies, such as Duecker et al. (2013), have also shown that the timing of stimulation during an ongoing process also matters. For these reasons, discomfort ratings for each stimulation site are acquired in the present study and will be used to test whether subjective discomfort accounts for differences in naming latencies. Moreover, the main effect of time-window will also be investigated in order to rule out any nonspecific effects of TMS that may be time-window specific. If there are truly nonspecific temporal effects of TMS, they should be present across stimulation sites as the effects are not specific to cortical stimulation. If there are site-specific nonspecific effects of TMS, they should scale with the subjective discomfort.

Taken together, the present study aims to conduct a modified replication of the Schuhmann et al. (2012) study while at the same time exploring alternative factors that may influence the findings such as naming latency variability and nonspecific effects of TMS. The present study emulates the experimental design and set-up of the original study by Schuhmann et al. (2012). At the same time, modifications are made in order to address the issues raised in the literature (Strijkers and Costa 2016). All differences between the present study and the original study (Schuhmann et al. 2012) can be found in Table 1.

(1) The pSTG & IFG stimulation sites from Schuhmann et al. (2012) are also stimulated in the present study. However, instead of their mMTG stimulation, pMTG is chosen as the third stimulation site. Considering pSTG stimulation in the original Schuhmann study did not interfere with phonological code retrieval, we reason that the specific stimulation site may have been involved in self-monitoring, leaving phonological code retrieval intact. As both pSTG and pMTG have been found to underlie phonological code retrieval (Indefrey and Levelt 2004), pMTG was chosen in order to interfere with phonological code retrieval.
(2) Since Schuhmann et al. (2012) along with other studies (Wheat et al. 2013; Shinshi et al. 2015; Zhang et al. 2018) have not found any effects for stimulation at 150-ms postpicture onset, the current study begins stimulation at 225-ms postpicture onset and the 5 time-windows are arranged to continuously cover the time range of 225–575 ms postpicture onset. Consult Table 1 for clarification.
(3) In addition to using pictures with monosyllabic labels as in the Schuhmann et al. (2012) study, the present study also comprises a second session utilizing pictures with bisyllabic labels in order to investigate how word length may modulate the spatiotemporal aspects of word production from the phonological code retrieval stage onward. Findings from the bisyllabic session are not directly comparable to the original study and serve as novel extension to the replication.

**Table 1:** Overview of the study by Schuhmann et al. (2012) and the present modified replication.

	Schuhmann et al. (2012)	Present study	
Stimulation Sites (left hemisphere)	mMTG^*^, pSTG, IFG	pMTG^*^, pSTG, IFG	
Time-windows	150^*^, 225, 300, 400^*^, 525	225, 300, 375^*^, 450^*^, 525	
Mean naming latencies of stimuli	Monosyllabic: 460 ms	Pre-test Monosyllabic: 600 ms Bisyllabic: 650 ms	Experiment Monosyllabic: 550 ms Bisyllabic: 600 ms
TMS sessions			
Monosyllabic stimuli	1 session for each stimulation site^*^ 3 sessions in total 60 trials per session 180 trials in total	1 session with all stimulation sites^*^ 1 session in total 180 trials per session 180 trials in total	
Bisyllabic stimuli^*^	Not performed	1 session with all stimulation sites 1 session in total 180 trials per session 180 trials in total	

Differences between the two studies are indicated with an asterisk.

In order to investigate the possibility that faster naming latencies may underlie early contributions of IFG, for the monosyllabic session, stimuli were pretested to have naming latencies closest to 600 ms. Stimuli for the bisyllabic session were centered ~650 ms based on the 50 ms per syllable processing time required during syllabification (Indefrey and Levelt 2004).

Overall, we expect pMTG stimulation to interfere with picture naming in an earlier time-window as compared with both pSTG and IFG. Similarly, we predict IFG stimulation to have the latest time-window effect among the 3 stimulation sites. Thus, with respect to the results of Schuhmann et al. (2012), we had the following hypotheses, which were tested in the present study:

(1) The stimulation effects in IFG will be replicated, but the time window of these stimulation effects should scale with speech onset latencies.
(2) The stimulation effects in pSTG ~400–450 ms will be replicated, but there should also be stimulation effects in posterior temporal lobe (pSTG, pMTG, or both) in the time window of phonological code retrieval (275–355 ms postpicture onset). Specifically, pMTG stimulation should interfere with phonological code retrieval in this time window.

## Materials and methods

### Participants

Twenty-four healthy, right-handed, native Dutch speakers (15 females, mean age = 22.5 ± 3.1 years) were recruited for the present study. All participants gave written informed consent and had no TMS contraindications. One participant was replaced due to issues with the audio recording and 2 participants were excluded from the analysis due to poor performance as reflected by error rates and delayed responses (>1,200 ms). All participants were compensated for their time. The study adhered to the Declaration of Helsinki and was approved by the competent reviewing authority (“Medical-Ethical Review Committee”), the “Commissie Mensgebonden Onderzoek regio Arnhem-Nijmegen”.

### Experimental design

The study entailed a 3 × 6 × 2 within-subject design with 3 stimulation sites (IFG, pMTG, and pSTG), 5 stimulation time-windows along with a no-stimulation condition (no TMS, 225, 300, 375, 450, and 525 ms), and 2 word lengths (monosyllabic and bisyllabic). The entire experiment was conducted at the Donders Center for Cognitive Neuroimaging (Radboud University) and comprised 3 sessions on 3 separate days. During the first session, participants came in for an MRI scan during which we acquired a *T*_1_-weighted structural scan. If participants already had a *T*_1_-weighted scan available, they were exempt from the scanning session. The following 2 TMS sessions were carried out identically except that monosyllabic stimuli were used in 1 session and bisyllabic stimuli in the other. The session order was counterbalanced across participants.

During each TMS session, participants named pictures while receiving online TMS to one of 3 stimulation sites. In each session, there was a total of 6 blocks and the TMS coil was moved to the subsequent stimulation site after 2 blocks. Each block consisted of 30 trials, meaning there were 60 trials per stimulation site and 180 per session. For each stimulation site, there was a corresponding set of 10 pictures that were presented 6 times each, once for each of the 5 stimulation time-windows along with the no TMS condition. Thus, each participant named the 10 pictures 6 times per stimulation site for a total of 60 trials. For all 3 stimulation sites, participants completed 180 trials in total per session. The order of stimulation sites was counterbalanced across participants and sessions. There was typically ~5–7 min between sites.

Prior to the first block of a new stimulation site, participants were familiarized with the set of pictures assigned to that particular stimulation site and participant via a sheet of paper containing the pictures and their corresponding labels. We also included 10 practice trials in which participants named each picture in the set once before beginning the first block of every new stimulation site. These 10 practice trials were not used in the analysis. Finally, upon completing 2 blocks of a particular stimulation site (typically about 15 min), participants were asked to rate the TMS-specific discomfort (annoyance, pain, muscle twitches) they experienced on a scale of 1–10 (Meteyard and Holmes 2018), which typically took them a couple of seconds.

All trials were recorded with a microphone for later offline analysis. Stimulus presentation, audio recording, and TMS stimulation were all controlled with Presentation (Neurobehavioral Systems, Inc., Albany, CA, USA). The trial timing was kept identical to the Schuhmann et al. (2012) study. A fixation cross was presented for a jittered duration between 5900 and 7900 ms, a blank screen for 100 ms followed by the target picture for 750 ms.

### Materials

#### Pretest

A total of 177 black and white line drawings were pretested, corresponding to 100 monosyllabic and 77 bisyllabic picture names. The drawings were taken from the picture database of the Max Planck Institute for Psycholinguistics in Nijmegen, the Netherlands, to remain consistent with previous studies (Schuhmann et al. 2009, 2012). Twenty participants (12 females, mean age = 23.4 ± 2.7 years) were recruited with the same criteria as mentioned in the Participants section and named all the pictures 6 times each as is the case in the main experiment. Half the participants started with the monosyllabic picture names, whereas the other half began with the bisyllabic picture names. Both the mono- and bisyllabic stimuli were presented in a pseudorandomized order to properly space out repetitions of the same picture. Participants recruited for the pretest did not take part in the main experiment.

A total of 60 pictures were selected to use for the TMS experiment. Because the aim of this experiment is to elucidate the effects of TMS on the time-course of word production, the thirty monosyllabic pictures with naming latencies closest to 600 ms and the thirty bisyllabic pictures with naming latencies ~650 ms were selected. Stimuli and stimuli parameters can be found in the Supplementary Materials Fig. S1 and Table S1, respectively.

### Experimental lists and design

For each syllabic type, the 30 pictures were grouped into 3 sets of 10 pictures each. Three sets of 10 pictures were used instead of 1 set of 10 pictures as in Schuhmann et al. (2012) to decrease the repetition count of each picture as well as to generalize the findings across a variety of controlled stimuli. The 3 picture sets were controlled for onset phonemes and consonant clusters as well as the semantic categories they belonged to. Thus, there was a similar proportion of animals, foods, and objects in each set. Each picture set was then assigned to a stimulation site in a counterbalanced fashion, further counterbalanced across participants using a lattice square design for each of the syllabic sets. That is, participants saw different pictures for each stimulated site and the site and picture set combination was different across participants. All pictures can be found in the Supplementary Materials Fig. S1.

**Fig. 2:**
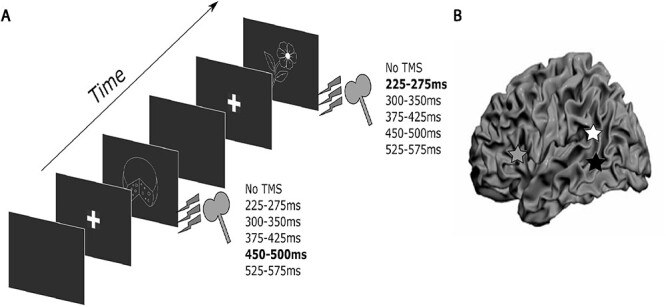
Experimental overview. **A**. Trial design. Chronometric online TMS was applied in one of five time-windows after picture onset to one of three stimulation sites (blocked design). Bolded times indicate an example stimulation time-window for the given trial. **B**. Stimulation sites (in Talairach coordinates) are represented by the grey star – left IFG (x, y, z = −49.9, 15.1, 20.8), white star – left pSTG (x, y, z = −56.1, −43.4, 14.2) and black star – left pMTG (x, y, z = −56.5, −44.7, 1.7).

### TMS protocol

Frameless infrared-based Neuronavigation (TMS Navigator, Localite, Sankt Augustin, Germany) based on individual anatomical *T*_1_-weigthed scans was used to navigate the TMS coil and to maintain its position over the respective target sites throughout the experiment. MR images were acquired using a rapid gradient echo (MPRAGE) sequence (1-mm isotropic). The mean Talairach coordinates from Schuhmann et al. were used for localizing the IFG (*x*, *y*, *z* = −49, 13, 26) and pSTG (*x*, *y*, *z* = −55, −44, 18) stimulation sites. On account of whether a more ventral position from the original pSTG site may disrupt phonological code retrieval, the pMTG (*x*, *y*, *z* = −55, −44, 9) stimulation site was localized just inferior to the pSTG stimulation site. Utilizing Localite, Tailarach coordinates were transformed into subject space and adjusted according to each individual’s anatomy. If transformed coordinates were positioned in a sulcus, then they were adjusted so as to stimulate the apex of the adjacent gyrus. For the temporal-lobe targets, adjustments were only made in the inferior–superior directions (i.e. a pMTG transform that was located in the superior temporal sulcus would be shifted inferiorly until it was on the apex of the MTG but no adjustments were made in the anterior–posterior directions). For IFG, the adjustment was made so that it was located superior to the ascending vertical ramus (Schuhmann et al. 2012). Post adjustment for individual anatomy, the mean Talairach coordinates for our study sample were: IFG (*x*, *y*, *z* = −49.9, 15.1, 20.8), pSTG (*x*, *y*, *z* = −56.1, −43.4, 14.2), and pMTG (*x*, *y*, *z* = −56.5, −44.7, 1.7).

Online triple-pulse TMS (tpTMS) at a frequency of 40 Hz and a stimulation intensity of 120% of the resting motor threshold (RMT) was used. Triple-pulse TMS with a frequency of 40 Hz was chosen for replication purpose (Schuhmann et al. 2012); however, it is also the most commonly used in chronometric TMS studies investigating picture naming (Schuhmann et al. 2009; Shinshi et al. 2015; Hämäläinen et al. 2018; Zhang et al. 2018). In both sessions, RMT was determined as the minimum intensity evoking a motor potential of at least 0.05 mV in the first dorsal interosseous muscle of the right hand in 50% of trials (Zhang et al. 2018). The mean stimulation intensity used during the experiment was 44.2 ± 7.2% (~67 A/μs) of maximum stimulator output for the first session and 44.7 ± 7.5% (~68 A/μs) for the second session. All TMS pulses were biphasic and applied with a figure 8 MagPro MC-B65-HO-8 coil (MagVenture, Farum, Denmark) connected to a Magpro-X-100 magnetic stimulator (MagVenture, Farum, Denmark). The coil was held tangentially to the skull and angled perpendicular to the stimulated gyrus. All participants wore earplugs throughout the duration of the experiment. The current TMS protocol adheres to international safety guidelines (Rossi et al. 2009, 2021).

Participants received triple-pulse TMS in 1 of 5 time-windows following picture onset. They were as follows: (i) 225–250–275 ms; (ii) 300–325–350 ms; (iii) 375–400–425 ms; (iv) 450–475–500 ms; and (v) 525–550–575 ms. Additionally, there was a sixth condition in which no TMS stimulation was applied. The 5 time-windows consecutively cover time points from 225 to 575 ms postpicture onset. In contrast to previous studies, we chose not to stimulate prior to 225 ms since no significant effects were ever reported in this early time-window (Schuhmann et al. 2009, 2012; Wheat et al. 2013; Shinshi et al. 2015; Hämäläinen et al. 2018; Zhang et al. 2018). Furthermore, the phonological processing stages targeted in this study are hypothesized to unfold after 200 ms (Indefrey and Levelt 2004; Indefrey 2011).

### Analysis

Naming latencies were determined offline using Praat (Boersma and van Heuven 2001; Boersma and Weenink 2019) blinded for stimulation site. Trials with an omitted or erroneous response were considered errors and excluded from all naming latency analyses. Trials in which the TMS pulses made it impossible to correctly determine word onset were also excluded from all naming latency analyses as well as the error analysis (Monosyllabic: 0.3%; Bisyllabic: 0.33%). In order to remain consistent with Schuhmann et al. (2012), all trials with naming latencies greater than 2SD from the participant’s mean (per stimulation site and time-window) were removed from naming latency analyses (Monosyllabic: 4.11%; Bisyllabic: 4.21%). In order to replicate the Schuhmann et al. (2012) study as best as possible, analyses were carried out to test for the effects of TMS pulse time within stimulation site for each word type. However, the present study utilizes mixed effects models to analyze the data as opposed to the ANOVAs used in the Schuhmann et al. (2012). All analyses were conducted in R (version 4.0.4; www.r-project.org). Furthermore, as a result of indirectly comparing across the 3 stimulation sites, a Bonferroni correction was applied for all analyses to better control for multiple comparisons. The alpha level for significance is therefore <0.016.

#### Discomfort ratings

TMS discomfort ratings were collected for each stimulation site (Meteyard and Holmes 2018). Since each participant provided one discomfort rating per stimulation site, an ANOVA was used to test for any differences in discomfort among the 3 stimulation sites as well as across sessions. Tukey’s test was then used to compare the 3 sites to one another while controlling for multiple comparisons.

#### Errors

Errors were analyzed using a mixed-effects logistic regression using the lmerTest package (Kuznetsova et al. 2017). The errors were analyzed for each word type and stimulation site. The model had errors as the dependent variable, TMS pulse time as the fixed factor, with the no-TMS condition as the reference level, and a by-participant random intercept. Items were originally also entered as random intercepts to remain consistent with the model used for the naming latency analysis (see Standard analysis of naming latencies section); however the model did not converge for the error analyses. As mentioned above, the alpha level for significance was set to <0.016.

#### Standard analysis of naming latencies

Naming latencies were analyzed using linear mixed-effects models with the lmerTest package in R (Kuznetsova et al. 2017). For the within stimulation site analyses, the models had the following parameters: TMS pulse time as a fixed factor, with the no-TMS condition as the reference level, and included by-item and by-participant random intercepts. As mentioned in the previous section, the alpha level for significance was lowered to <0.016.

#### Baseline response-locked analysis

The following analysis was performed separately for each word type. Each participant’s naming latency in the no-TMS condition per picture was used as the participant’s picture-specific baseline naming latency (PP-BNL). The PP-BNL was used to account for variability across participants and pictures and reflects the typical naming speed for a given participant and picture should no TMS interference occur. In order to have a baseline response-locked stimulation time point for each trial (word), each participant’s picture-specific baseline (i.e. no-TMS condition) naming latency was subtracted from the TMS stimulation time point (relative to stimulus onset). Since the TMS stimulation comprised 3 pulses, the time of the last pulse was used (e.g. if stimulation on a given trial occurred at 300, 325, and 350 ms, then 350 ms was used as the TMS stimulation time point). To illustrate, in trial *t*, a participant is presented with picture *C*, for which the PP-BNL is 600 ms (naming latency in the no-TMS condition). In trial *t*, the TMS stimulation occurred in the 300–350 ms time-window relative to stimulus-onset, so the corresponding response-locked time point of trial *t* is −250 ms (350–600 ms), meaning that the last TMS stimulation in trial *t* was delivered 250 ms prior to the participant’s PP-BNL for picture *C*.

**Fig. 3:**
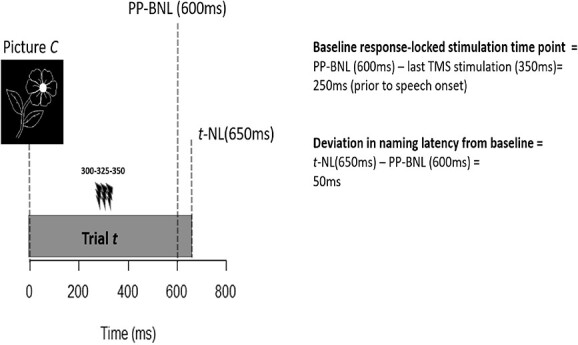
Visual schematic of baseline response-locked analysis. PP-BNL = Participant and picture-specific baseline naming latency, t-NL = trial-specific naming latency.

Next, the trial-specific deviations in naming latencies from the above-mentioned PP-BNL were calculated. Continuing from the above example, if this participant had a trial-specific naming latency (*t*-NL) of 650 ms for trial *t*, then the deviation in naming latency would be 50 ms (650–600 ms). Hence, TMS stimulation 250 ms prior to the typical naming time (PP-BNL) resulted in a 50-ms increase in naming latency for trial *t*. Figure 3 illustrates the aforementioned calculations.

From the above procedure, the data of each participant result in an irregularly sampled time series for each picture, with baseline response-locked time points on the *x*-axis and deviation in naming latencies on the *y*-axis (see Fig. 6). To estimate a continuous regular time series for each participant, a moving mean with a Gaussian filter was used to approximate the mean value for each time point. A 100-ms time-window was utilized and the Gaussian filter ensured that values in the center of the window had more weight. The window was moved in steps of 25 ms. To accept a time-window estimate, we set a minimum of 7 data points contributing to the mean value.

This procedure resulted in a regular time series for each picture for each participant. Note that different groups of pictures were assigned to different stimulation sites. Therefore, the following step necessitated averaging across these groups of pictures in order to obtain time series for each participant and stimulation site, and subsequently averaging across participants to obtain a site-specific time series. For statistical inference of these timeseries data, we used a cluster-based permutation analysis for each site-specific time series whereby the deviations in naming latencies from baseline (in ms) were compared with a theoretical control condition consisting of zeros (i.e. TMS had no effect). The cluster-based permutation analysis was performed using the clusterperm.lmer function in the permutes package (Voeten 2019, 2021). The function utilizes an alpha-level significance threshold equivalent to *P* < 0.05.

#### Nonspecific TMS effects

Finally, main effects of TMS pulse time, stimulation site, and subjective discomfort ratings on naming latencies were analyzed to determine whether nonspecific effects of TMS might be present. Since the interest is on the nonspecific effects of TMS, the No TMS condition was not included in this analysis. Naming latencies were analyzed using linear mixed-effects models (lmerTest package, Kuznetsova et al. 2017). Three models were used, all of which included by-item and by-participant random intercepts. First, to test for a main effect of TMS pulse time, the model had naming latencies as the dependent variable and TMS pulse time as the fixed factor, with sequential contrasts. For effects of stimulation site, the model had naming latencies as the dependent variable and stimulation site as the fixed factor, with pMTG acting as the reference level. Lastly, to test for the effect of subjective discomfort, a third model with both stimulation site and subjective discomfort as fixed factors was constructed. Importantly, this third model was compared with the second model using an ANOVA in order to determine whether adding subjective discomfort ratings significantly improves the model fit. Again, the alpha level for significance was lowered to <0.016.

## Results

### Errors

Error rates were comparable across the 2 sessions (Monosyllabic: 4.11%; Bisyllabic: 4.21%). Table 2 provides a statistical summary of the error analysis. During monosyllabic naming, there were no significant effects of TMS pulse time within any of the stimulation sites. However, during bisyllabic naming, stimulating IFG at 450 ms resulted in significantly more errors as compared with the no-TMS condition. No other effect reached significance during bisyllabic naming.

**Table 2:** Inferential statistics for errors.

	*B*	SE	*z*	*P*
Monosyllabic session				
*IFG*				
Intercept	−3.598	0.423	−8.51	**<0.001**
No-TMS vs. 225 ms	0.583	0.481	1.211	0.229
No-TMS vs. 300 ms	0.266	0.508	0.525	0.6
No-TMS vs. 375 ms	1.038	0.453	2.291	0.022
No-TMS vs. 450 ms	1.041	0.453	2.297	0.022
*pMTG*				
Intercept	−3.858	0.481	−8.021	**<0.001**
No-TMS vs. 225 ms	<0.001	0.641	0.000	1.000
No-TMS vs. 300 ms	−0.522	0.738	−0.707	0.479
No-TMS vs. 375 ms	0.641	0.568	1.081	0.280
No-TMS vs. 450 ms	0.730	0.558	1.307	0.191
*pSTG*				
Intercept	−3.676	0.432	−8.502	**<0.001**
No-TMS vs. 225 ms	−0.188	0.609	−0.309	0.757
No-TMS vs. 300 ms	0.533	0.522	1.022	0.307
No-TMS vs. 375 ms	0.968	0.490	1.975	0.048
No-TMS vs. 450 ms	1.109	0.482	2.299	0.0215
Bisyllabic session				
*IFG*				
Intercept	−3.698	0.436	−8.471	**<0.001**
No-TMS vs. 225 ms	−0.146	0.541	−0.270	0.787
No-TMS vs. 300 ms	−0.146	0.541	−0.270	0.787
No-TMS vs. 375 ms	0.250	0.499	0.501	0.616
No-TMS vs. 450 ms	1.4408	0.433	3.328	**<0.001**
*pMTG*				
Intercept	−3.230	0.390	−8.431	**<0.001**
No-TMS vs. 225 ms	−0.238	0.490	−0.486	0.627
No-TMS vs. 300 ms	−0.238	0.490	−0.486	0.627
No-TMS vs. 375 ms	−0.540	0.530	−1.018	0.308
No-TMS vs. 450 ms	−0.225	0.490	−0.460	0.645
*pSTG*				
Intercept	−3.428	0.401	−8.542	**<0.001**
No-TMS vs. 225 ms	0.216	0.466	0.464	0.643
No-TMS vs. 300 ms	0.311	0.458	0.679	0.497
No-TMS vs. 375 ms	0.656	0.435	1.506	0.132
No-TMS vs. 450 ms	−0.418	0.542	−0.771	0.441

IFG = inferior frontal gyrus, pMTG = posterior middle temporal gyrus, pSTG = poterior superior temporal gyrus. Significant effects are *P* < 0.016.

Bold values represent a significant effect.

**Table 3:** Inferential statistics for naming latencies.

	B	SE	*t*	*P*
Monosyllabic Session				
*IFG*				
Intercept	556.343	12.887	43.172	**<0.001**
No-TMS vs. 225 ms	−8.85	6.966	−1.27	0.204
No-TMS vs. 300 ms	−5.949	6.966	−0.854	0.393
No-TMS vs. 375 ms	2.905	7.066	0.411	0.681
No-TMS vs. 450 ms	28.543	7.025	4.063	**<0.001**
*pMTG*				
Intercept	542.714	14.764	36.758	**<0.001**
No-TMS vs. 225 ms	−5.438	5.68	−0.957	0.339
No-TMS vs. 300 ms	−0.591	5.667	−0.104	0.917
No-TMS vs. 375 ms	11.884	5.73	2.074	0.038
No-TMS vs. 450 ms	27.321	5.71	4.785	**<0.001**
*pSTG*				
Intercept	545.626	10.99	49.65	**<0.001**
No-TMS vs. 225 ms	−12.404	5.495	−2.257	0.024
No-TMS vs. 300 ms	−10.687	5.546	−1.927	0.054
No-TMS vs. 375 ms	20.479	5.561	3.682	**<0.001**
No-TMS vs. 450 ms	5.689	5.577	1.02	0.308
Bisyllabic session				
*IFG*				
Intercept	607.98	14.273	42.597	**<0.001**
No-TMS vs. 225 ms	−12.872	6.841	−1.882	0.06
No-TMS vs. 300 ms	−9.655	6.836	−1.412	0.158
No-TMS vs. 375 ms	0.321	6.87	0.047	0.963
No-TMS vs. 450 ms	6.224	7.067	0.881	0.379
*pMTG*				
Intercept	598.678	12.951	46.226	**<0.001**
No-TMS vs. 225 ms	−18.026	6.872	−2.623	**0.009**
No-TMS vs. 300 ms	−18.163	6.856	−2.649	**0.008**
No-TMS vs. 375 ms	−1.036	6.832	−0.152	0.88
No-TMS vs. 450 ms	−3.978	6.936	−0.574	0.566
*pSTG*				
Intercept	581.518	12.75	45.608	**<0.001**
No-TMS vs. 225 ms	−10.291	6.37	−1.616	0.106
No-TMS vs. 300 ms	−3.627	6.393	−0.567	0.571
No-TMS vs. 375 ms	5.056	6.422	0.787	0.431
No-TMS vs. 450 ms	18.619	6.364	2.925	**0.003**

IFG = inferior frontal gyrus, pMTG = posterior middle temporal gyrus, pSTG = poterior superior temporal gyrus. Significant effects are *P* < 0.016. Bold values represent a significant effect.

### Naming latencies

Although the stimuli had been pretested so that monosyllabic stimuli had an average naming latency of 600 ms and the bisyllabic stimuli of 650 ms, the mean naming latencies in the TMS study turned out to be ~550 ms for monosyllabic words and 600 ms for bisyllabic words. It may be the case that participants were anticipating the TMS stimulation and therefore were more alert and responded faster than during the pretest where no such anticipation was present. Even though naming was faster than pretested, the ~50 ms gap between mono- and bisyllabic naming latencies was still present, meaning that our hypothesis concerning word length remained testable. However, as a result of faster naming latencies, it became apparent that many monosyllabic items were named prior to TMS stimulation when stimulation occurred in the 525–575 time-window (41.97%). This issue was also present during the bisyllabic session (22.8%). These trials are problematic since they do not reflect TMS effects, yet removing them skews the data for the 525 time-window and hence affects the model outputs. We therefore removed this time-window from the analysis. For a full analysis including the 525 time-window, see Supplementary Materials Fig. S2. This issue did not affect the remaining time-windows.

**Fig. 4:**
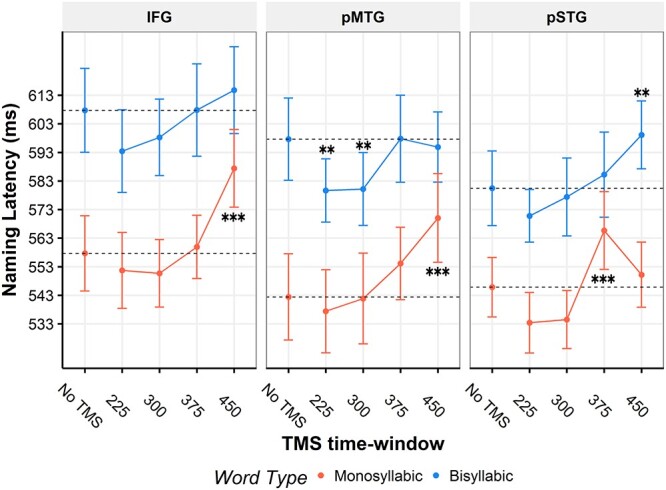
Naming latency results. Mean naming latencies across TMS time-windows for each stimulation site. Error bars indicate the standard error of the mean. Asterisks indicate significant effects relative to the no-TMS condition. ^**^P < 0.01, ^***^P < 0.001. IFG = inferior frontal gyrus, pMTG = posterior middle temporal gyrus, pSTG = posterior superior temporal gyrus.

For the within stimulation site analyses, Fig. 4 shows the mean naming latencies across time-points for each respective stimulation site. For all 3 stimulation sites, descriptively, a general trend of facilitation (e.g. compare the 225-ms time window to the no-TMS condition) from TMS is present during the earlier time-windows, whereas a general trend toward interference is present in later time-windows. During monosyllabic picture naming, TMS to left IFG significantly increased naming latencies when stimulated in the 450-ms window (*P* < 0.001)**.** IFG stimulation did not yield any significant effects for bisyllabic naming. pMTG stimulation also increased naming latencies when stimulation was applied at 450 ms (*P* < 0.001) during monosyllabic naming but not during bisyllabic naming. There were, however, facilitatory effects during the earlier time-windows of 225 ms (*P* = 0.008) and 300 ms (*P* = 0.007) when pMTG was stimulated during bisyllabic picture naming. Finally, pSTG stimulation resulted in an interference effect on naming latencies when stimulated at 375 ms (*P* < 0.001) for monosyllabic naming. For bisyllabic naming, TMS to pSTG increased naming latencies during the 450-ms time-window (*P* = 0.003). A statistical summary of the findings can be found in Table 3.

### Baseline response-locked analysis

**Fig. 5:**
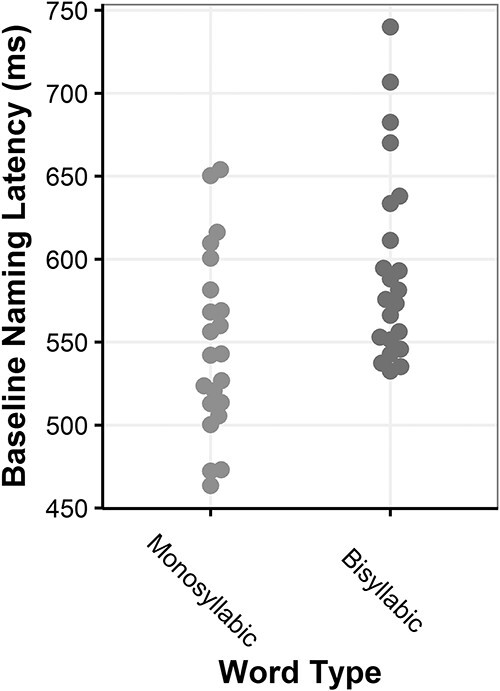
Baseline Naming Latencies. Participants' baseline naming latencies during the no-TMS condition. Each dot represents one participant.

Figure 5 shows participants’ naming latencies in the baseline (no TMS) condition averaged over pictures and stimulation sites. As mentioned earlier, it is evident that naming latencies across participants varied substantially even after careful selection of materials for which naming latencies were predetermined to be homogeneously ~600 ms. This means that TMS stimulation during, for example, the 300-ms time-window might disrupt a given processing stage in 1 participant and completely miss it in another, simply due to this individual variability in naming speed.

With varying naming latencies, the temporal estimates of the initial stages of word-production might be more consistent when measured from stimulus-onset, whereas the temporal estimates of later stages may be more consistent when measured from the response. The more processing moves forward in the chain of events, the greater the chance that different participants will be at different stages of the word production process. For this reason, the baseline response-locked analysis was performed in order to better understand effects at late stages of word production.

A cluster-based permutation analysis was performed to examine whether certain time points had naming latencies that significantly deviated from baseline naming latencies. Generally speaking, the same qualitative trend is observed across all stimulation sites and word types: facilitation effects at earlier time points and interference effects closer to speech onset. IFG stimulation led to interference in naming latencies when stimulation occurred between 150 and 50 ms prior to speech onset for monosyllabic naming, whereas this was limited to 125–100 ms prior to speech for bisyllabic naming. Stimulating pMTG resulted in significant interference in naming latencies between 125 and 25 ms prior to speech onset during monosyllabic naming and 125–75 ms during bisyllabic naming. Furthermore, pMTG stimulation also resulted in significant facilitation between 300 and 225 ms prior to speech onset, roughly corresponding to the facilitation effects found in the standard analysis. Finally, stimulating pSTG resulted in significant facilitation in naming latencies between 275 and 225 ms and significant interference between 150 and 50 ms prior to speech onset for monosyllabic naming. During bisyllabic naming, pSTG stimulation interfered with naming between 100 and 50 ms prior to speech onset.

### Discomfort ratings

The ANOVA revealed a main effect for stimulation site (*F*(2, 63) = 14.43, *P* < 0.001). IFG discomfort ratings (mean = 5.06, median = 5, range = 1.66–8.33) were found to be significantly higher compared with those of pMTG (mean = 3.65, median = 3.33, range = 1–7, *t*(21) = 3.517, *P* = 0.002) and pSTG (mean = 2.95, median = 2.5, range = 1.3–6.66, *t*(21) = 5.275, *P* < 0. 001). There was no significant difference between pSTG and pMTG discomfort ratings (*t*(21) = 1.758, *P* = 0.19). Moreover, there was also a significant effect in discomfort rating between sessions (*t*(21) = −2.69, *P* = 0.008), where the second session had lower discomfort ratings as compared with the first.

### Nonspecific effects of TMS

In line with this descriptive trend of facilitation-like effects in earlier time-windows and interference-like effects in later ones, the model investigating main effects of TMS pulse time did yield a significant effect but only for the 300- vs. 375-ms time-window (*P* < 0.001). Specifically, as compared with the 300-ms time-window, stimulating any of the 3 regions at 375-ms poststimulus onset resulted in significantly longer naming latencies. Furthermore, the model for stimulation site also yielded an effect. Stimulating IFG (*P* < 0.001) as compared with pMTG resulted in longer naming latencies. This effect was not present for pSTG stimulation as compared with pMTG. The last model with stimulation site and subjective discomfort as fixed factors, IFG stimulation was still significant, although to a lesser degree (*P* = 0.01) and subjective discomfort was also found to be significant (*P* < 0.001). Upon preforming an ANOVA, it became clear that adding subjective discomfort as a fixed factor significantly improved the model fit (*P* < 0.001). A statistical summary can be found in Table 4.

## Discussion

The present study investigated the temporal contribution of the left IFG, pMTG, and pSTG to picture naming. We sought to replicate previous findings of Schuhmann et al. (2012) while addressing issues regarding inconsistencies with the literature. Moreover, we also conducted a baseline response-locked analysis in order to control for the variation in naming latencies among participants under the rationale that the timing of processing stages is likely to increasingly diverge across participants, as well as from established temporal estimates, as time unfolds. Therefore, the standard, stimulus-locked analysis should be more informative of earlier time-window effects, whereas response-locked analysis should be more informative for the late stages. Finally, nonspecific effects of TMS were also investigated.

The most consistent observation is that TMS has the most perturbing effects, as indexed by a slowing of naming latency, when delivered late in the planning phase, close to articulatory onset. This effect was observed for all 3 stimulation targets. This could suggest an overall higher susceptibility for perturbation, perhaps with less opportunity for compensation, late in the naming processing, or it could be indicative of a nonspecific effect of TMS. Nonetheless, irrespective of the general slowing for late TMS, we observed specific effects dependent on stimulation site. Below, we first discuss our findings per region separately, before providing an integrative view over our findings.

**Fig. 6:**
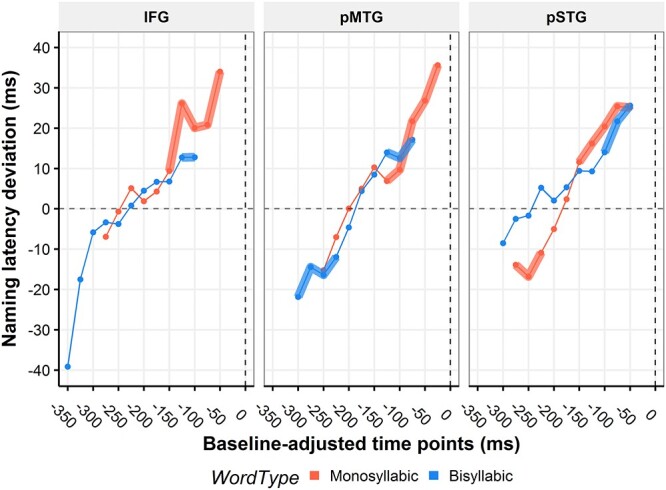
Baseline Adjusted Response-Locked analysis. Response-locked time series for each stimulation site. Bolded lines represent significant clusters (*P* < 0.05) yielded from the cluster-based permutation analysis. IFG = inferior frontal gyrus, pMTG = posterior middle temporal gyrus, pSTG = posterior superior temporal gyrus.

### Temporal dynamics of word production

#### Inferior frontal gyrus

During monosyllabic naming, IFG seems to contribute to monosyllabic picture naming ~450–500 ms. Previous studies have found IFG to be causally involved in earlier time-windows of 200–250 ms (Hämäläinen et al. 2018), 225–275 ms (Wheat et al. 2013; Zhang et al. 2018), 300–350 ms (Schuhmann et al. 2009, 2012; Wheat et al. 2013; Shinshi et al. 2015; Hämäläinen et al. 2018; Zhang et al. 2018), and 400–450 ms (Zhang et al. 2018). IFG involvement at 450–500 ms in the present study, therefore, seems rather late. The time window also appears to be late compared with the I&L model estimate for syllabification (~355–455 ms). In a language mapping study conducted by Sollmann et al. (2017), however, stimulation of the opercular part of IFG resulted in more performance errors (thought to reflect articulatory planning) as compared with other error types when stimulation commenced at 200-, 400-, and 500-ms postpicture onset. TMS to IFG in the present study may have therefore disrupted articulatory processing rather than syllabification. The standard analysis showed no IFG effects during the bisyllabic naming session.

**Fig. 7:**
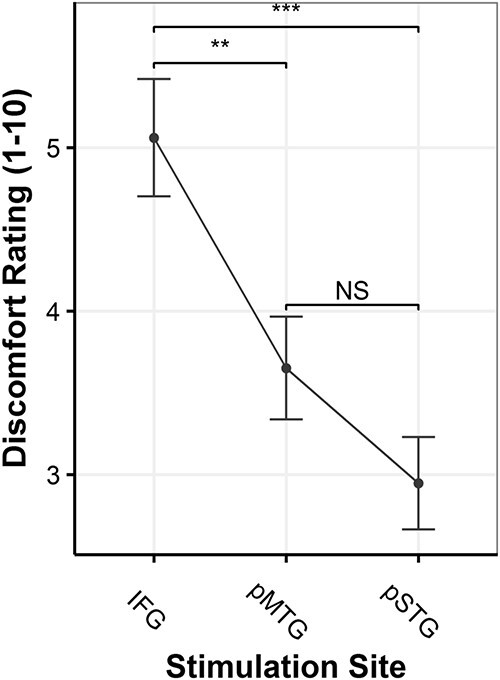
Mean discomfort ratings collapsed across word type and session. Error bars indicate the standard error of the mean. IFG = inferior frontal gyrus, pMTG = posterior middle temporal gyrus, pSTG = posterior superior temporal gyrus. Discomfort was rated on a scale of 1 to 10. Higher ratings indicate more discomfort.

Analyzing the data in a response-locked fashion showed IFG stimulation to significantly increase naming latency from baseline at 150–50 ms prior to speech during monosyllabic naming, which corresponds to the 450–500 ms time-window in the standard analysis. During the bisyllabic session, IFG stimulation significantly increased naming latency deviations between 125 and 100 ms prior to speech. Importantly, as suggested by the mean baseline naming latencies shown in Fig. 5, stimulation at 450 ms (latest time-window) fell within 100 ms before baseline response onset for about two thirds of the trials during monosyllabic naming. However, during bisyllabic naming, the same stimulation only fell within 100 ms before typical response onset in ~10% of the trials. Hence, possible interference due to stimulation shortly before articulation could not have been detected for bisyllabic words in the standard analysis. In contrast, the 125–100 ms stimulation time point (prior to speech-onset) in the baseline response-locked analysis was able to account for longer latency bisyllabic words, so that significant interference by stimulation shortly before articulation onset could be detected. In other words, what is a nonsignificant trend in Fig. 4 becomes significant in Fig. 6. This pattern of results confirms our assumption that a baseline response-locked analysis should be superior to the standard analysis for the detection of stimulation effects at late processing stages.

In sum, IFG seems to significantly contribute to naming at 150–50 ms (monosyllabic words) and 125–100 ms (bisyllabic words) prior to speech onset. Based on the 600-ms speech onset latency assumed by the I&L model, these intervals would correspond to an interval of 450–550 ms postpicture onset that is rather late compared with the estimate of the model (355–455 ms). However, when comparing our results from the monosyllabic naming session to those of Schuhmann et al. (2012), although the timing relative to stimulus-onset does not correspond, the timing relative to speech-onset does seem to fall in-line. In our study, IFG stimulation showed effects 150–50 ms prior to speech onset corresponding to 400–500 ms from stimulus onset (taking the mean naming latency of ~550 ms in the no-TMS condition to be the baseline). In the Schuhmann et al. (2012) study, the baseline naming latency was ~450 ms, meaning that their IFG effect at 300–350 ms also coincides with the interval of 150–100 ms prior to speech onset.

**Table 4:** Main and nonspecific effects for naming latencies.

**Main effect of TMS pulse time**				
**Condition/parameter**	**B**	**SE**	***t***	***P***
Intercept	572.034	10.677	53.576	**<0.001**
300 ms vs. 225 ms	1.78	2.892	0.616	0.538
375 ms vs. 300 ms	15.581	2.912	5.351	**<0.001**
450 ms vs. 375 ms	6.859	2.941	2.332	0.0197
**Main effect of Stimulation site**				
**Condition/parameter**	**B**	**SE**	***t***	***P***
Intercept	568.844	10.766	52.837	**<0.001**
IFG vs. pMTG	13.31	2.542	5.237	**<0.001**
pSTG vs. pMTG	−4.679	2.543	−1.840	0.066
**Main effect of Stimulation site and Subjective Discomfort**				
**Condition/parameter**	**B**	**SE**	***t***	***P***
Intercept	554.022	11.527	48.065	**<0.001**
IFG vs. pMTG	7.584	2.944	2.576	**0.01**
pSTG vs. pMTG	−1.843	2.644	−0.697	0.486
Subjective Discomfort	4.054	1.056	3.838	**<0.001**
**ANOVA**				
**Model**	**# Parameters**	**AIC**	***P***	
Stimulation site	6	55,008		
Stimulation site + Subjective discomfort	7	54,995	**<0.001**	

IFG = inferior frontal gyrus, pMTG = posterior middle temporal gyrus, pSTG = poterior superior temporal gyrus. Significant effects are *P* < 0.016. Bold values represent a significant effect.

It thus seems that our first hypothesis was confirmed: the time window of stimulation effects in IFG indeed depends on speech onset latencies. The time window of stimulation inducing an IFG effect was shifted by about the increase in onset latencies compared with Schuhmann et al. (2012). We can, therefore, interim conclude that IFG stimulation affects a late process. However, it may well be that this late process is articulatory planning rather than syllabification or phonetic encoding and that the effect is caused by nonspecific effects of stimulation rather than cortical stimulation, as discussed in more detail below.

#### Posterior middle temporal gyrus

pMTG stimulation resulted in interference effects in the 450–500 ms time-window during monosyllabic naming and facilitatory effects in early time-windows of 225–275 and 300–350 ms during bisyllabic naming. Studies using chronometric TMS to investigate the role of non-IFG regions in picture naming are much fewer. Only one study used online TMS to probe pMTG during picture naming and only in a time-window of −100 ms (prior to picture-onset) to 200 ms postpicture onset (Acheson et al. 2011). The authors did find this stimulation to significantly delay picture naming; however, it is important to note that their stimulation site was placed at the border of pMTG and mMTG; hence, stimulation possibly affected the earlier lemma selection stage (Indefrey and Levelt 2004). In the I&L model, pMTG is thought to be involved in phonological code retrieval ~275–355 ms. Our monosyllabic effect at 450–500 ms is late compared with the model, but our effects on naming bisyllabic words at 225–275 and 300–375 ms overlap with the model’s estimates.

In the baseline response-locked analysis, pMTG stimulation during monosyllabic naming showed similar results to those of IFG but with a slight shift, namely significant increases in naming latencies when stimulated 125–25 ms prior to speech onset. This time-window corresponds to the 450–500 ms time-window from the standard analysis. Similar to our findings on IFG, there was also a late effect for pMTG stimulation during bisyllabic naming (125–75 ms prior to speech onset) that was not present in the standard analysis. An earlier facilitation effect was also observed when pMTG was stimulated at 300–225 ms before speech onset. This roughly corresponds to the facilitation effects found in the 225–350 ms time-window from the standard analysis.

Thus, pMTG tends to present a similar issue as IFG, namely overlap in later time-windows between the 2 baseline response-locked analyses (125–25 and 125–75 ms prior to speech onset) and the standard analysis during monosyllabic naming (450–500 ms), but no such effect during bisyllabic naming in the standard analysis.

The pMTG site was originally chosen to attempt to interfere with phonological code retrieval thought to take place ~275–355 ms poststimulus onset. Although not present in the monosyllabic session, effects between 225 and 350 ms were observed in the bisyllabic session, which match the theorized time-window. Furthermore, based on the mean naming latency of ~600 ms in the no-TMS condition, the early significant effects from the baseline response-locked analysis (300–225 ms prior to speech onset) correspond to 300–355 ms poststimulus onset, which is also in line with the temporal estimate for phonological code retrieval. However, it is unclear why this effect is only present during bisyllabic naming. One potential reason could lie in the lack of sensitivity of our exploratory baseline response-locked analysis for earlier processing stages. As can be seen in Fig. 6, pMTG also shows a facilitation effect ~250 ms prior to speech onset, but there is insufficient data to look at effects further back in time.

#### Posterior superior temporal gyrus

pSTG stimulation replicated the interference effect at 400–500 ms reported by Schuhmann et al. (2012). We found pSTG stimulation to interfere with the production of monosyllabic words when delivered at 375–425 ms (150–50 prior to baseline naming onset) and to interfere with the production of bisyllabic words when delivered at 450–500 ms (100–50 prior to baseline naming onset). In the study by Sollmann et al. (2017), pSTG stimulation was found to predominantly produce hesitation errors (defined as a delay of more than 200 ms) when stimulation started at 300-, 400-, and 500-ms postpicture onset. This falls roughly in line with the current results and speaks to pSTG’s role in later processing stages.

The later time-window in the bisyllabic session may reflect the later word onsets, shifting in time appropriately. These findings also fall in line with pSTG’s role in self-monitoring as assumed by Indefrey and Levelt (2004). Importantly, the baseline response-locked analysis showed that TMS delivered to pSTG also had an earlier facilitation effect at 275–225 ms prior to baseline speech onset which was not observed in the standard analysis. It is worth noting that pSTG stimulation in the 225–275 and 300–350 ms time-windows did show an effect in the standard analysis, but it did not surpass the alpha level we adopted to control for multiple comparisons.

Overall, the late effects in pSTG are consistent with a role of this region in self-monitoring as assigned in the I&L model. However, similar to our cautionary remark in the IFG effect above, we think that alternative explanations for this late stimulation effect cannot be ruled out (see below).

The facilitation effect at 275–225 ms prior to speech onset during monosyllabic naming overlaps in time with the pMTG effect at 300–225 ms prior to speech onset during bisyllabic naming. These effects in part confirm our second hypothesis that TMS stimulation in the posterior temporal lobe should not only have a late effect but also affect naming responses when delivered in the earlier time window of phonological code retrieval assumed by the I&L model (275–355 ms postpicture onset). Nonetheless, there are open issues. Effects of stimulation in the 2 posterior temporal regions differed between shorter and longer picture names and, most importantly, we found facilitation effects rather than the expected interference.

#### Facilitation vs. interference

Typically, online TMS protocols tend to interfere with ongoing activity. Unexpected improvements in performance from such protocols have therefore been appropriately termed “paradoxical facilitation” (Bergmann and Hartwigsen 2021). As the name might suggest, the underlying mechanisms as to how TMS causes such facilitation effects remains largely unclear although some suggestions have been put forward (see Bergmann and Hartwigsen 2021, for an overview): TMS as adding neural noise to ongoing neural activity (Miniussi et al. 2010; Abrahamyan et al. 2011); TMS interfering in a brain area that is not relevant to the current task (Walsh et al. 1998; Luber and Lisanby 2014); or via disinhibition of a connected node in a network (Sandrini 2011).

Finally, facilitation effects may also arise from nonspecific effects of TMS (which will be discussed in the subsequent section). Of these possibilities, optimal noise or disinhibition of a connected brain region are likely to underlie the facilitation effects observed in the present study, but this account remains speculative.

### Neuromodulation or nonspecific effects of TMS?

#### Stimulation site and subjective discomfort

One important question to ask with any online TMS experiment is whether the findings reflect true neural effects or whether they are a consequence of nonspecific effects induced by the TMS stimulation. As mentioned earlier, Meteyard and Holmes (2018) found that discomfort varies across the scalp depending on the underlying anatomy and that subjective discomfort ratings were able to predict reaction time differences in previously published studies (Holmes and Meteyard 2018). Generally, the more uncomfortable the stimulation is perceived to be, the longer reaction times. In the present study, discomfort was significantly higher for IFG stimulation as compared with pMTG and pSTG; however, there was no difference between pMTG and pSTG. This falls in line with frontal areas being more uncomfortable, as more muscles are present in this region. As for pMTG and pSTG, their scalp locations are in the same general vicinity, and therefore, the lack of a significant difference in perceived discomfort also makes sense. Interestingly, IFG stimulation also resulted in significantly longer naming latencies as compared with pMTG, whereas no difference in naming latencies was present when comparing pSTG to pMTG stimulation. Therefore, some correspondence between site-specific subjective discomfort and naming latencies seems to exist; the site with significantly higher discomfort ratings also resulted in significantly longer naming latencies. Furthermore, when comparing the mixed effects models, the model that included discomfort ratings as a fixed factor yielded a significant improvement in model fit, indicating that subjective discomfort did account for our findings on top of just stimulation site.

#### Time-windows

Nonspecific TMS effects can also differ depending on the timing of TMS stimulation during an ongoing process. Duecker and Sack (2013) investigated the effects that sham stimulation (i.e. stimulation that mimics real TMS but does not stimulate the cortex) has on performance and found that sham stimulation alone can speed up or slow down response times (Duecker et al. 2013; Duecker and Sack 2015). In their study, the researchers performed a single pulse chronometric TMS experiment where they stimulated the vertex (often used as a control region in TMS studies) with real and sham TMS. Their results showed that both sham and real TMS had effects on the subsequent reaction times as compared with a no TMS condition despite the fact that both sham stimulation and real TMS stimulation over the vertex should not have affected cognitive processes. Our current results show a similar pattern of facilitation effects in earlier time-windows and interference effects in later time-windows to their sham condition during an angle judgment task (Duecker et al. 2013). Interestingly, in the present study, a significant effect of TMS pulse time was present when the 375-ms time-windows was compared with the 300-ms time-window, irrespective of stimulation site or word type. Although the other contrasts were not significant, a clear linear trend remarkably similar to that found in Duecker et al. (2013) is present. Therefore, it is likely that nonspecific effects of TMS are also present in our study.

Although nonspecific effects of TMS are present, it does not exclude the presence of genuine “cortical effects” of TMS. However, it does mean that the results of the present study should be interpreted in the context of these nonspecific effects, as it remains unclear to what degree nonspecific or cortical stimulation is responsible for the behavioral outcomes. The addition of a nonlanguage stimulation sites may help untangle neuromodulatory vs. nonspecific contributions of TMS.

### Limitations of chronometric TMS in overt production tasks

TMS can interfere with articulation by 2 possible mechanisms. First, the stimulation of the muscle directly, especially with high frequencies, can lead to tetanus-like effects which may delay articulation. This is a muscle-specific effect. Second, stimulating the muscle will cause afferent signals to be relayed to the brain. How exactly these afferent signals may affect articulatory motor planning just prior to articulatory onset is unclear.

According to an influential model of speech motor control, the DIVA model (Guenther and Vladusich 2012), the somatosensory state map is a crucial part of motor planning. In order to properly coordinate the movement of articulators, their initial positions must be known. The DIVA model begins at the level of phonetic encoding, and thus, somatosensory information becomes crucial for motor planning ~450 ms according to the I&L model (Indefrey and Levelt 2004). Prior to this, it may be possible for the articulators to stabilize after stimulation and therefore not have any effect once motor planning begins.

Although sparse, there are a few studies that directly investigated brain activity elicited by neuromuscular stimulation. Wegrzyk et al. (2017), for example, electrically stimulated the right triceps surae (calf muscle) and recorded the subsequent brain activity using fMRI. Electric stimulation of the muscle resulted in the activation of M1, S1, S2, premotor cortex, putamen, thalamus, caudate nucleus, cerebellum, and many other brain regions. These regions comprise the same motor network recruited during articulatory motor planning (Guenther and Vladusich 2012). Regardless of how exactly muscular stimulation-induced brain activity may interact with ongoing articulatory motor planning, the vast amount of cortical activation elicited by muscular stimulation cannot be ignored.

## Conclusion

In summary, the present study utilized online chronometric TMS to probe 3 different brain regions at various time-windows during picture naming. The study sought to replicate and extend the findings of Schuhmann et al. (2012), as well as elucidate empirical inconsistencies of such paradigms in the literature. Overall, IFG, pMTG, and pSTG stimulation all significantly affected naming ~150–50 ms prior to baseline speech onset. Posterior temporal lobe stimulation in an earlier time-window of 225–350 ms facilitated naming. These findings are in line with assumed functional roles of IFG and pSTG in later processing stages (phonetic encoding, articulatory planning, self-monitoring) as well as a functional role of the posterior temporal lobe in phonological code retrieval. In general, the present study replicates the findings of the original Schuhmann et al. (2012) study. First, as in the original study, we find temporally specific contributions of the stimulated brain regions. Although the absolute timings differ, the relative timings adjusted for variations in naming latencies appear to be consistent. TMS chronometry therefore seems to be valid method for probing the temporal contributions of specific brain regions. However, the results should always be interpreted in relative terms and take into account nonspecific effects of TMS.

Importantly, in particular with respect to the effect of stimulation in later time windows, we remain critical in interpreting the results and raise issues regarding the nonspecific effects of TMS, the effects of individual variability in naming latencies, and muscle stimulation confounds. Thus, in addition to providing theoretically relevant results, the present study’s also offers new ways of approaching the data and raises key considerations for interpreting results from chronometric TMS studies investigating overt production. Specifically, response-locked analyses are recommended for chronometric TMS studies that have time-windows close to speech onset or where variation in individual naming latencies is present. Lastly, readers and researchers interpreting results of chronometric TMS studies should take care to consider alternative methodological explanations and not assume that behavioral findings are solely driven by cortical stimulation.

## Supplementary Material

SupplementaryMaterialsFinal_AdrianJodzio_bhad081

## Data Availability

The data will be made available on the Donders Repository at https://data.donders.ru.nl/.

## CRediT authors statement

Adrian Jodzio: Conceptualization, Data curation, Formal analysis, Investigation, Methodology, Project administration, Visualization, Writing – original draft, Writing – review & editing. Vitoria Piai: Conceptualization, Formal analysis, Investigation, Resources, Software, Supervision, Validation, Writing – review & editing. Lennart Verhagen: Formal analysis, Methodology. Ian Cameron: Conceptualization, Data curation, Investigation, Methodology, Project administration, Resources, Software, Supervision, Validation, Writing – review & editing. Peter Indefrey: Conceptualization, Formal analysis, Funding acquisition, Investigation, Project administration, Resources, Supervision, Validation, Writing – review & editing.
